# Liposome-Mediated Inhibition of Inflammation by Hydroxycitrate

**DOI:** 10.3390/nano10102080

**Published:** 2020-10-21

**Authors:** Antonio Vassallo, Valentina Santoro, Ilaria Pappalardo, Anna Santarsiero, Paolo Convertini, Maria De Luca, Giuseppe Martelli, Vittoria Infantino, Carla Caddeo

**Affiliations:** 1 Department of Science, University of Basilicata, Viale dell’Ateneo Lucano 10, 85100 Potenza, Italy; antonio.vassallo@unibas.it (A.V.); ilaria.pappalardo@unibas.it (I.P.); anna.santarsiero@unibas.it (A.S.); paolo.convertini@uky.edu (P.C.); maria.deluca@unibas.it (M.D.L.); giuseppe.martelli@unibas.it (G.M.); 2 Department of Pharmacy, University of Salerno, Via Giovanni Paolo II 132, 84084 Salerno, Italy; valesantoro86@gmail.com; 3 KAMABIO Srl, Via Al Boschetto 4/B, 39100 Bolzano, Italy; 4 ALMACABIO Srl, C/so Italia 27, 39100 Bolzano, Italy; 5 Department of Scienze della Vita e dell’Ambiente, Sezione di Scienze del Farmaco, University of Cagliari, Via Ospedale 72, 09124 Cagliari, Italy

**Keywords:** hydroxycitrate, liposomes, macrophages, inflammation, antioxidant

## Abstract

Hydroxycitrate (HCA), a main organic acid component of the fruit rind of *Garcinia cambogia*, is a natural citrate analog that can inhibit the ATP citrate lyase (ACLY) enzyme with a consequent reduction of inflammatory mediators (i.e., nitric oxide (NO), reactive oxygen species (ROS), and prostaglandin E_2_ (PGE_2_)) levels. Therefore, HCA has been proposed as a novel means to prevent, treat, and ameliorate conditions involving inflammation. However, HCA presents a low membrane permeability, and a large quantity is required to have a biological effect. To overcome this problem, HCA was formulated in liposomes in this work, and the enhancement of HCA cell availability along with the reduction in the amount required to downregulate NO, ROS, and PGE_2_ in macrophages were assessed. The liposomes were small in size (~60 nm), monodispersed, negatively charged (−50 mV), and stable on storage. The in vitro results showed that the liposomal encapsulation increased by approximately 4 times the intracellular accumulation of HCA in macrophages, and reduced by 10 times the amount of HCA required to abolish LPS-induced NO, ROS, and PGE_2_ increase. This suggests that liposomal HCA can be exploited to target the citrate pathway involved in inflammatory processes.

## 1. Introduction

Lipopolysaccharide (LPS)-activated or classically activated macrophages acquire an inflammatory phenotype that triggers a series of metabolic changes, such as raised oxidative phosphorylation, reduced fatty acid synthesis, and production of the inflammatory mediators nitric oxide (NO), reactive oxygen species (ROS), prostaglandin E_2_ (PGE_2_), IL-1β, and other cytokines [1].

More recently, it has been discovered that metabolic shifts of activated macrophages, in many cases shared with cancer cells [2], also involve the Krebs cycle rewiring, leading to the accumulation of citrate, succinate, and fumarate [3]. In LPS- or TNFα/IFNγ-activated macrophages, the mitochondrial citrate carrier (CIC) exports citrate from the mitochondria to the cytosol where the ATP citrate lyase (ACLY) enzyme catalyzes its conversion into oxaloacetate (OAA) and acetyl-CoA. OAA is then reduced to malate, which in turn is converted to pyruvate. This reaction produces Nicotinamide adenine dinucleotide phosphate (NADPH), a source of reducing equivalents required for ROS and NO synthesis [4]. ROS generation from NADPH oxidase complex in the presence of NADPH and O_2_ represents a critical step in the inflammatory response [5]. The synthesis of NO inflammatory mediator from L-arginine, catalyzed by inducible nitric oxide synthase (iNOS), also requires NADPH and molecular oxygen [6]. In contrast, cytosolic acetyl-CoA is converted to malonyl-CoA, which is a substrate for fatty acid biosynthesis including arachidonic acid, the precursor of prostaglandins. Among them, prostaglandin E2 (PGE_2_) is a key modulator of inflammation and innate immunity and plays a crucial role in inflammatory diseases [7].

Of note, ACLY mRNA levels are increased in Behçet’s syndrome, a multisystemic inflammatory disease, and both CIC and ACLY protein levels are raised in children with Down syndrome, a genetic disorder showing some inflammatory phenotypic aspects [8,9].

Remarkably, the inhibition of CIC or ACLY activity leads to a reduction of PGE_2_ secretion in LPS-, TNFα-, and IFNγ-induced macrophages. Furthermore, upon ACLY or CIC gene silencing or CIC or ACLY activity inhibition, a significant lowering in ROS and NO levels has been reported in activated macrophages [10,11,12].

In light of the reported observations, the inhibition of the citrate export pathway might reduce several macrophage mediators, thus being effective in suppressing inflammatory processes. Among ACLY inhibitors, (3R,5S)-rel-5-[6-(2,4-dichlorophenyl)hexyl]tetrahydro-3-hydroxy-2-oxo-3-furanacetic acid (SB-204990) and hydroxycitrate (HCA) significantly reduce NO, ROS, and PGE_2_ pro-inflammatory signals in activated macrophages [13].

SB-204990 and HCA led to a decrease of both NO and ROS levels and oxidative stress in cells from children with Down Syndrome [8]. SB-204990 is a cell-permeable pro-drug much better absorbed than HCA [14]. However, HCA shows the meaningful advantage of being a natural product; it is a main organic acid component of the fruit rind of *Garcinia gummi-gutta*, commonly known as *Garcinia cambogia* [15]. Because of its low membrane permeability, like all highly hydrophilic metabolites, a large quantity must be used in order to observe a biological effect. In our previous experiments, an amount of 500 μM HCA was found to be the minimum amount needed to induce an anti-inflammatory effect [12]. In this work, with the aim of reducing the therapeutic dose of HCA needed to counteract inflammation, the use of nanocarriers was attempted. Nanocarriers offer an innovative approach to drug delivery through different administration routes, providing a number of benefits that include cargo protection, modified pharmacokinetics and distribution, increased dose delivery to target sites, enhanced drug transport through biological membranes, and prolonged or controlled drug release. Among nanocarriers, liposomes are one of the most promising. Due to their structure and composition, liposomes can carry both hydrophilic and lipophilic compounds, while being biocompatible and safe. For these reasons, in this work we investigated whether liposomal encapsulation could improve HCA cell availability and thus reduce the amount of HCA required to exert its anti-inflammatory effect through the inhibition of NO, ROS, and PGE_2_.

## 2. Materials and Methods

### 2.1. Materials

Hydroxycitrate (HCA), L-glutamine, penicillin/streptomycin solution, lipopolysaccharide from *Salmonella enterica* serotype typhimurium (LPS), Roswell Park Memorial Institute 1640 (RPMI 1640), and phorbol 12-myristate 13-acetate (PMA) were purchased from Sigma-Aldrich (St. Louis, MO, USA). Soy lecithin was purchased from Galeno (Carmignano, Italy).

### 2.2. Liposome Preparation and Characterization

For the preparation of HCA-loaded liposomes (Lip-HCA), 120 mg/mL of soy lecithin and 2 mg/mL of HCA were weighed in a glass vial, dispersed in water, and sonicated (15 cycles, 5 s on and 2 s off; 13 μm of probe amplitude) with a high-intensity ultrasonic disintegrator (Soniprep 150, MSE Crowley, London, UK).

The average diameter and polydispersity index (PI; a measure of the size distribution width) of the vesicles were determined by dynamic light scattering using a Zetasizer Nano ZS (Malvern Instruments, Worcestershire, UK). Zeta potential was estimated using the Zetasizer Nano ZS by means of the Mixed Mode Measurement Phase Analysis Light Scattering (M3-PALS) technique, which measures the particle electrophoretic mobility. The samples (*n* > 6) were diluted with water (1:100) and analyzed at 25 °C. For comparative purposes, empty liposomes (i.e., without HCA) were also prepared and characterized. The stability of liposomes was evaluated by long-term stability tests, i.e., by analyzing vesicle average diameter, PI, and zeta potential over three months at 25 °C.

### 2.3. Cell Culture and Treatments

Human monoblastic leukemia U937 cell line (Interlab Cell Line Collection (ICLC) HTL94002) was grown in suspension in RPMI 1640 medium supplemented with 10% (*v*/*v*) fetal bovine serum, 2 mM L-glutamine, 100 U/mL penicillin, and 100 μg/mL streptomycin at 37 °C in 5% CO_2_ in a water-saturated atmosphere. Pro-monocytic U937 cells were differentiated to macrophages by 10 ng/mL phorbol 12-myristate 13-acetate (PMA). Then, U937/PMA cells were treated with 50 or 500 µM free hydroxycitric acid (f-HCA) or HCA-loaded liposomes (Lip-HCA) and, where indicated, stimulated with 100 ng/mL of lipopolysaccharide from *Salmonella enterica* serotype typhimurium (LPS).

### 2.4. Cytotoxicity Assay

The effects of f-HCA and Lip-HCA on U937/PMA cell proliferation were determined using a Millipore Scepter™ handheld automated cell counter (Merck Millipore, Darmstadt, Germany) 72 h after incubation, according to the manufacturer’s instructions. Briefly, cells were cultured into 96-well plates (5 × 10^4^ cells/well) and treated with f-HCA and Lip-HCA diluted with RPMI medium to achieve the desired concentrations (50 or 500 µM). After 72 h, U937/PMA cells were collected in 1.5 mL microfuge tubes and counted.

### 2.5. ROS, NO, and PGE_2_ Detection

To evaluate reactive oxygen species (ROS) and nitric oxide (NO) levels, U937/PMA cells were triggered by 100 ng/mL of LPS in the presence or absence of f-HCA and Lip-HCA (50 or 500 µM). After 24 h, ROS and NO concentrations were measured by using 6-carboxy-2’,7’-dichlorodihydrofluorescein diacetate (DCF-DA, Thermo Fisher Scientific, San Jose, CA, USA) and 4-amino-5-methylamino-2’,7’-difluorofluorescein diacetate (DAF-FM diacetate, Thermo Fisher Scientific), respectively. Briefly, cells were collected in tubes and counted. After centrifugation, the cell pellet was resuspended in PBS to have 10^5^ cells/100 μL. Then, 10 µM DCF-DA or 2.24 µM DAF-FM diacetate were added. After 30 min of incubation in the dark at 37 °C, a 100 μL portion of sample was transferred to triplicate groups of wells on a black microtiter plate and the fluorescence was revealed using a GloMax plate reader (Promega, Madison, WI, USA).

For prostaglandin E_2_ (PGE_2_) detection, U937/PMA cells were treated with 50 or 500 μM f-HCA or Lip-HCA for 1 h before triggering inflammation with LPS. After 48 h, PGE_2_ levels were quantified in cell culture medium by using a DetectX^®^ Prostaglandin E_2_ High Sensitivity Immunoassay Kit (Arbor Assays, Ann Arbor, MI, USA) according to the manufacturer’s protocol. Specifically, 100 μL of standards and samples were put into a clear microtiter plate coated with an antibody direct to mouse IgG. A PGE_2_–peroxidase conjugate was added. The binding reaction, initiated by the addition of a monoclonal antibody to PGE_2_, lasted overnight. The day after, the plate was washed and the substrate, which reacted with the bound PGE_2_–peroxidase conjugate, was added. After 20 min of incubation, the reaction was stopped and absorbance at 450 nm was measured using a GloMax plate reader (Promega). Values of each sample were normalized to protein content.

### 2.6. Cellular Uptake of HCA by HPLC–Tandem Mass Spectrometry

To quantify the cellular uptake of HCA, U937/PMA cells were seeded into 6-well plates at a density of 2.5 × 10^5^ cells/well, treated with 50 or 500 μM of HCA (f-HCA or Lip-HCA) for 24 h, and pelleted at 1200 rpm for 5 min. Supernatants were removed. To extract HCA, 500 μL of 40% MeOH with 0.1% (*v*/*v*) formic acid were added to the cells, which were lysed by sonication (1 min on/1 min off, 10 min total), mixed, and placed on ice for 15 min. Lysates were then centrifuged at 1500 rpm for 5 min. Supernatants were collected for LC–MS analysis. To ensure that all of the HCA was extracted from cells, another amount of 250 μL of 40% MeOH with 0.1% (*v*/*v*) formic acid was added to the cell pellets and suspended again. After 15 min, on ice, the suspensions were centrifuged at 14,000 rpm for 5 min, and all supernatants were pooled together [16,17]. The supernatants were dried with vacuum-centrifugation using a Concentrator plus (Eppendorf AG, Hamburg, Germany), the solid residues were dissolved in 100 μL of 40% MeOH with 0.1% (*v*/*v*) formic acid, and 10 μL aliquots were injected in a UPLC–ESI–Qtrap system. The protein concentration in each sample was determined by the Bradford assay [18], and cell lysates were checked to verify success of the lyses step and to normalize multiple samples for side-by-side comparison. Mass spectrometry-based analyses were carried out to evaluate the amount of HCA in differently treated cells. A NexeraX2 UPLC (Shimadzu, Kyoto, Japan) coupled with an ABSciex API6500 Q-Trap spectrometer (Foster City, CA, USA) was used. The analyses were performed by using a multiple reaction monitoring (MRM) protocol, operating in negative ion mode [19]. After chromatography and mass spectrometry parameter optimization, the following conditions were selected: chromatographic analysis was performed on a Luna reverse phase C18 column (100 × 2 mm, 2.6 μm; Phenomenex, Torrance, CA, USA), using ammonium acetate 5 mM in H_2_O (Eluent A) and ammonium acetate 5 mM in MeOH (Eluent B) as mobile phase. The elution conditions were as follows: linear gradient from 2% to 6% of B in 3 min, followed by a faster gradient until 95% of B in 7 min. The flow rate was 0.2 mL/min, and the injection volume was 10 μL. The MS parameters were as follows: precursor ion 207 *m*/*z*, product ions 127 and 189 *m*/*z*, declustering potential −50 eV, entrance potential −9 eV, collision energy −12 eV, and collision cell exit potential −20 eV [20].

### 2.7. Statistical Analysis

Statistical significance of differences was determined using the Student’s *t*-test. Results are shown as means ± SD of, at least, three independent experiments. Differences were considered as significant (#, * *p* < 0.05, # vs. control, * vs. LPS), very significant (##, ** *p* < 0.01), and highly significant (###, *** *p* < 0.001).

## 3. Results

### 3.1. Liposomal Formulation

HCA-loaded liposomes were prepared by a simple method involving the sonication of the phospholipid (soy lecithin) and HCA in water. To evaluate the effect of the encapsulation of HCA on the vesicle arrangement, empty liposomes were also prepared and characterized. As shown by the results summarized in Table 1, empty liposomes displayed small size (63 nm), good homogeneity (PI 0.24), and highly negative zeta potential (−49 mV). HCA had a negligible effect on the analyzed vesicles’ characteristics (*p* > 0.05; Table 1).

The stability of the prepared liposomes was evaluated by monitoring the average diameter, PI, and zeta potential over three months of storage. The results showed no significant variations (*p* > 0.05) of the parameters examined, which indicates a good stability of the vesicle formulations.

### 3.2. Effect of HCA-Loaded Liposomes on Cell Count

First, we evaluated the cytotoxicity of HCA-loaded liposomes (Lip-HCA) by cell counting. To this end, U937/PMA cells were treated with 50 or 500 µM Lip-HCA or free-HCA (f-HCA), used as control, for 72 h. As illustrated in Table 2, neither f-HCA nor Lip-HCA significantly altered cell number at the tested concentrations.

### 3.3. Effect of HCA-Loaded Liposomes on PGE_2_ Production

**Figure 1:**
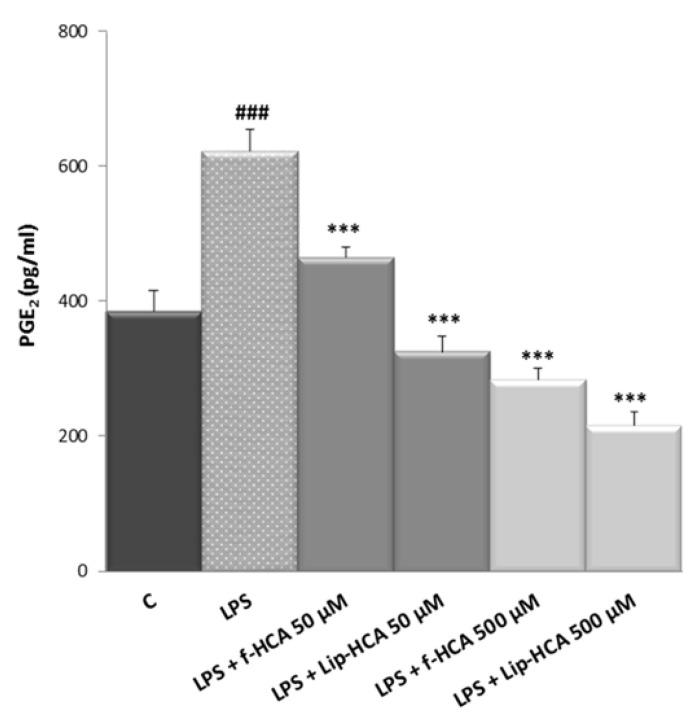
Effect of free HCA (f-HCA) and HCA-loaded liposomes (Lip-HCA) on prostaglandin E_2_ (PGE_2_) production. U937/PMA cells were triggered by LPS in the presence or absence of Lip-HCA and f-HCA (50 or 500 µM). After 48 h, PGE_2_ levels were quantified. Results are presented as means ± SD from three independent experiments. ###, *** *p* < 0.001, ^#^ vs. control (C, untreated cells), * vs. LPS.

Having proven that Lip-HCA did not strongly reduce, but only slightly at the highest concentration, the number of U937/PMA cells, we evaluated their anti-inflammatory activity by testing their inhibitory effects on the production of inflammatory mediators. Since it has been reported that the inhibition of ACLY activity by 500 μM f-HCA reduces PGE_2_ production [12], which is the main product of the COX-2 pathway in inflammatory conditions, we assessed the effect of Lip-HCA treatment on PGE_2_ secretion. f-HCA was used as control in U937/PMA cells triggered by LPS. As shown in Figure 1, when LPS-activated cells were treated with 500 µM Lip-HCA, PGE_2_ levels were reduced 24% more than with f-HCA (*p* < 0.01). Surprisingly, 50 µM Lip-HCA completely abolished the LPS effect, and PGE_2_ secretion was significantly lowered (about 30%) as compared to f-HCA (Figure 1). These results suggest that Lip-HCA controls PGE_2_ secretion at a concentration 10 times lower than that of f-HCA commonly used.

### 3.4. Effect of HCA-Loaded Liposomes on NO and ROS Production

**Figure 2:**
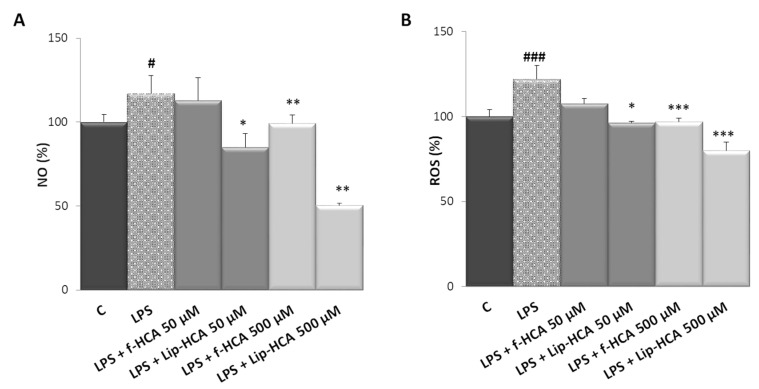
Effect of free HCA (f-HCA) and HCA-loaded liposomes (Lip-HCA) on nitric oxide (NO) (**A**) and reactive oxygen species (ROS) (**B**) production. U937/PMA cells were triggered by 100 ng/mL of LPS in the presence or absence of Lip-HCA and f-HCA (50 or 500 µM). After 24 h, NO and ROS levels were quantified. Results are presented as means ± SD from three independent experiments. #, * *p* < 0.05, ** *p* < 0.01, ###, *** *p* < 0.001, # vs. control (C, untreated cells), * vs. LPS.

To further explore the anti-inflammatory effect of Lip-HCA, we monitored the levels of inflammatory mediators closely associated with cellular energy status 24 h after treatment. NO, a mediator and regulator of inflammatory response, is synthesized from L-arginine in a reaction catalyzed by inducible nitric oxide synthase (iNOS). The conversion of L-arginine to NO and L-citrulline requires NADPH as substrate. Infantino et al. [13] reported that the inhibition of ACLY activity by 500 μM f-HCA caused a great reduction in NO levels in LPS-activated M1 macrophages. As shown in Figure 2A, the treatment with Lip-HCA, at both tested concentrations, reduced NO levels as compared to LPS-induced cells. However, it should be noted that an amount of 50 μM HCA was able to restore the control levels of NO only when encapsulated in liposomes. Indeed, an amount of 50 μM f-HCA was not able to lower NO production (Figure 2A). Subsequently, the effect of Lip-HCA on ROS production was tested using f-HCA as control in U937/PMA cells triggered by LPS. ROS are generated by the NADPH oxidase complex in the presence of molecular oxygen and NADPH [21]. Under the same experimental conditions, and at a concentration of 500 µM, both f-HCA and Lip-HCA reduced ROS levels compared to LPS-activated macrophages. Interestingly, at a concentration of 50 µM, only Lip-HCA completely abolished the ROS production induced by LPS treatment (Figure 2B). Altogether these data demonstrate that liposome encapsulation decreases by 10 times the HCA amount required to abrogate the surplus of both NO and ROS levels synthesized upon LPS addition.

### 3.5. Intracellular Uptake of HCA

**Figure 3:**
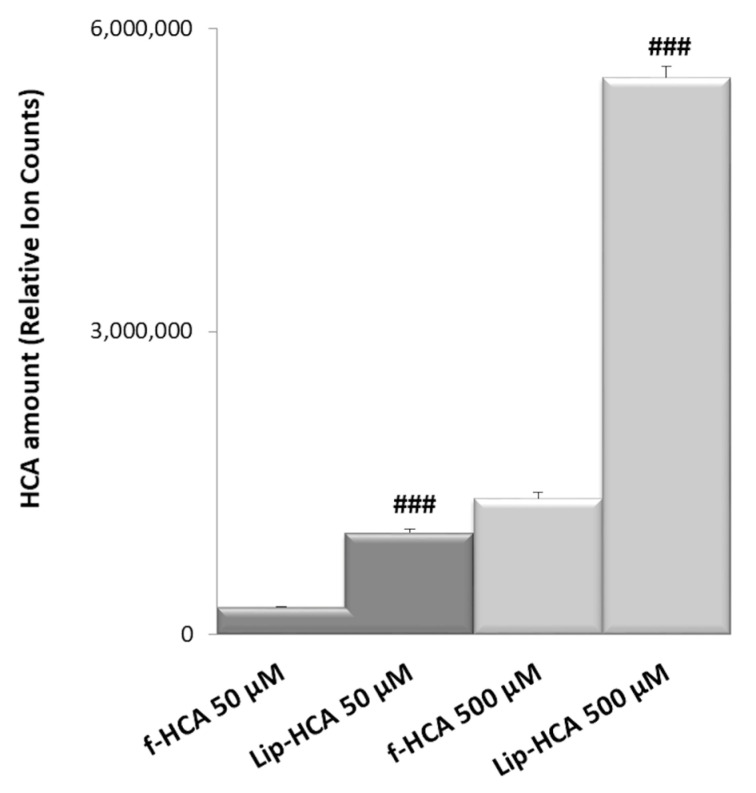
Relative amounts of HCA accumulated in U937/PMA cells after 24 h of exposure to free HCA (f-HCA) and HCA-loaded liposomes (Lip-HCA) (50 and 500 μM). Results are presented as means ± SD (*n* = 3). ### *p* < 0.001 (50 μM f-HCA vs. 50 μM Lip-HCA, and 500 μM f-HCA vs. 500 μM Lip-HCA).

In order to evaluate the HCA cell intake after cell incubation with the free compound or with its liposomal formulation, LC–MS analyses were performed. To obtain a good resolution and sensitivity for the HCA detection, different chromatographic conditions, in terms of eluent composition and pH, were tested. LC–MS analyses can provide information on the amount of HCA within cells upon different treatments. U937/PMA cells were incubated with f-HCA and Lip-HCA at two different concentrations (50 and 500 μM) for 24 h. The results confirmed that the use of the liposomal formulation clearly increased the amount of intracellular HCA, at both tested concentrations (Figure 3). The amount of HCA detected within the cells was higher (~3.7-fold) when HCA was formulated in liposomes, rather than in solution at 50 µM (Figure 3). When HCA was loaded in liposomes (500 µM), its relative abundance was ~4.1-fold higher than in solution.

## 4. Discussion

HCA has long been known for its anti-obesity activity by inhibiting ACLY, a key enzyme of fatty acid biosynthesis [22]. More recently, ACLY has been found upregulated by LPS as well as pro-inflammatory cytokines, and the inhibition of its activity by HCA suppresses NO, ROS, and PGE_2_ production in M1 macrophages [12]. Therefore, the anti-inflammatory activity is a novel health benefit of HCA, thus widening its range of therapeutic properties. Since HCA shows a high hydrophilicity and low membrane permeability, a large amount is needed in order to observe a biological effect. To overcome this problem, liposomes were used in this study to load and deliver HCA to the cells, and to ascertain whether the nanoencapsulation might lower the amount required to exert an anti-inflammatory activity.

Nanodelivery systems, among which liposomes are considered one of the most successful, have been shown to boost bioavailability and efficacy of their cargo, as they facilitate the entry through biological barriers, while avoiding the metabolic modifications that can reduce absorption [23,24]. Liposomes have been used to deliver both synthetic and natural drugs, and thanks to their flexible physico-chemical and biophysical properties, they can be easily modified to address different delivery issues [25,26,27].

Recently, HCA has been nanoencapsulated in solid lipid nanoparticles (SLNs). Ezhilarasi et al. reported that HCA-loaded SLNs exhibited a 2-fold higher bioavailability than non-encapsulated HCA and a 1.3-fold higher bioavailability than microparticles, which was ascribed to smaller particle size, longer residence time, and controlled release of SLNs [28]. In that study, HCA was used as a model hydrophilic drug to investigate the efficiency of SLN to load hydrophilic compounds, and to evaluate the effect of micro- and nanoparticles on the bioavailability of such compounds, while its biological activities were not assessed.

To the best of our knowledge, this is the first study that reports the nanoencapsulation of HCA in liposomes and the evaluation of the enhancement of its anti-inflammatory activity in macrophages thanks to the delivery by a vesicular carrier system. Our results point to the key role of the nanocarrier in the intracellular uptake process, as liposomes enhanced (by approximately 4 times) the accumulation of HCA within the cells, where the anti-inflammatory activity is exerted. Indeed, NO, ROS, and PGE_2_ production was suppressed by liposomal HCA at a concentration 10 times lower than that of non-encapsulated HCA. Therefore, a small amount of liposomal HCA at the same time inhibits the production of multiple inflammatory mediators, making it very effective in blocking inflammatory responses. Moreover, liposomal HCA, by targeting immunometabolism through ACLY metabolic enzyme, could represent an innovative approach to many pathological conditions, since inflammation underlies countless diseases. Finally, as ACLY is also overexpressed in a wide variety of tumors, such as hepatocellular carcinoma [2], it is reasonable to suppose that liposomal HCA might be useful for the treatment of cancer, maybe in combination with other drugs.

## 5. Conclusions

The results of the present work demonstrate that the prepared liposomes were remarkably effective in delivering the natural molecule HCA, as they promoted its entry in macrophages where the citrate pathway was inhibited, with a consequent suppression of the inflammatory mediators NO, ROS, and PGE_2_. This suggests the use of liposomal HCA to target inflammatory diseases and highlights the need for further research, such as targeted in vivo tests, to validate the in vitro antioxidant/anti-inflammatory potential of HCA. Indeed, the discovery that liposomes potentiate HCA biological activities allows for the development of novel and more effective approaches based on nanotechnologies to treat a variety of conditions that involve excessive inflammation.

## Figures and Tables

**Table 1:** Characteristics of empty liposomes and hydroxycitrate (HCA)-loaded liposomes: intensity-weighted mean hydrodynamic diameter, polydispersity index (PI), and zeta potential (ZP). Each value represents the mean ± SD, *n* > 10; ^§^ SD for PI values was always < 0.02.

	Mean Diameter	PI ^§^	ZP
	(nm ± SD)		(mV ± SD)
Empty Liposomes	63 ± 4.4	0.24	−49 ± 13.8
HCA Liposomes	65 ± 9.1	0.26	−59 ± 6.3

**Table 2:** Effect of free HCA (f-HCA) and HCA-loaded liposomes (Lip-HCA) on U937/PMA cell number. Cells were treated with f-HCA or Lip-HCA (50 or 500 µM) for 72 h. Results are presented as percentage (%) ± SD from three independent experiments.

Cell Number (% ± SD)			
	0 µM	50 µM	500 µM
f-HCA	100 ± 3.6	103.5 ± 3.1	98.7 ± 2.5
Lip-HCA	100 ± 5.3	118.8 ± 2.4	92.3 ± 2.8

## References

[1] O’Neill L.A., Hardie D.G. (2013). Metabolism of inflammation limited by AMPK and pseudo-starvation. Nature.

[2] Todisco S., Convertini P., Iacobazzi V., Infantino V. (2019). TCA Cycle Rewiring as Emerging Metabolic Signature of Hepatocellular Carcinoma. Cancers.

[3] Ryan D.G., O’Neill L.A.J. (2017). Krebs cycle rewired for macrophage and dendritic cell effector functions. FEBS Lett..

[4] Iacobazzi V., Infantino V. (2014). Citrate—New functions for an old metabolite. Biol. Chem..

[5] Lee I.T., Yang C.M. (2012). Role of NADPH oxidase/ROS in pro-inflammatory mediators-induced airway and pulmonary diseases. Biochem. Pharmacol..

[6] Lauterbach M.A., Hanke J.E., Serefidou M., Mangan M.S.J., Kolbe C.C., Hess T., Rothe M., Kaiser R., Hoss F., Gehlen J. (2019). Toll-like Receptor Signaling Rewires Macrophage Metabolism and Promotes Histone Acetylation via ATP-Citrate Lyase. Immunity.

[7] Park J.Y., Pillinger M.H., Abramson S.B. (2006). Prostaglandin E2 synthesis and secretion: The role of PGE2 synthases. Clin. Immunol..

[8] Convertini P., Menga A., Andria G., Scala I., Santarsiero A., Castiglione Morelli M.A., Iacobazzi V., Infantino V. (2016). The contribution of the citrate pathway to oxidative stress in Down syndrome. Immunology.

[9] Santarsiero A., Leccese P., Convertini P., Padula A., Abriola P., D’Angelo S., Bisaccia F., Infantino V. (2018). New Insights into Behcet’s Syndrome Metabolic Reprogramming: Citrate Pathway Dysregulation. Mediat. Inflamm..

[10] Infantino V., Convertini P., Cucci L., Panaro M.A., Di Noia M.A., Calvello R., Palmieri F., Iacobazzi V. (2011). The mitochondrial citrate carrier: A new player in inflammation. Biochem. J..

[11] Infantino V., Iacobazzi V., Menga A., Avantaggiati M.L., Palmieri F. (2014). A key role of the mitochondrial citrate carrier (SLC25A1) in TNFalpha- and IFNgamma-triggered inflammation. Biochim. Biophys. Acta.

[12] Infantino V., Iacobazzi V., Palmieri F., Menga A. (2013). ATP-citrate lyase is essential for macrophage inflammatory response. Biochem. Biophys. Res. Commun..

[13] Infantino V., Pierri C.L., Iacobazzi V. (2019). Metabolic Routes in Inflammation: The Citrate Pathway and its Potential as Therapeutic Target. Curr. Med. Chem..

[14] Abramson H.N. (2011). The lipogenesis pathway as a cancer target. J. Med. Chem..

[15] Semwal R.B., Semwal D.K., Vermaak I., Viljoen A. (2015). A comprehensive scientific overview of Garcinia cambogia. Fitoterapia.

[16] Caddeo C., Nacher A., Vassallo A., Armentano M.F., Pons R., Fernandez-Busquets X., Carbone C., Valenti D., Fadda A.M., Manconi M. (2016). Effect of quercetin and resveratrol co-incorporated in liposomes against inflammatory/oxidative response associated with skin cancer. Int. J. Pharm..

[17] Pham A., Bortolazzo A., White J.B. (2012). Rapid dimerization of quercetin through an oxidative mechanism in the presence of serum albumin decreases its ability to induce cytotoxicity in MDA-MB-231 cells. Biochem. Biophys. Res. Commun..

[18] Bradford M.M. (1976). A rapid and sensitive method for the quantitation of microgram quantities of protein utilizing the principle of protein-dye binding. Anal. Biochem..

[19] Chirollo C., Vassallo A., Dal Piaz F., Lamagna B., Tortora G., Neglia G., De Tommasi N., Severino L. (2018). Investigation of the Persistence of Penicillin G and Dihydrostreptomycin Residues in Milk of Lactating Buffaloes (*Bubalus bubalis*) Using Ultra-High-Performance Liquid Chromatography and Tandem Mass Spectrometry. J. Agric. Food Chem..

[20] Pandey R.C.P., Kumar B., Srivastva B., Aravind A., Rameshkumard S.K.B. (2015). Simultaneous determination of multi-class bioactive constituents for quality assessment of Garcinia species using UHPLC–QqQLIT–MS/MS. Ind. Crops Prod..

[21] Babior B.M. (2004). NADPH oxidase. Curr. Opin. Immunol..

[22] Shara M., Ohia S.E., Yasmin T., Zardetto-Smith A., Kincaid A., Bagchi M., Chatterjee A., Bagchi D., Stohs S.J. (2003). Dose- and time-dependent effects of a novel (−)-hydroxycitric acid extract on body weight, hepatic and testicular lipid peroxidation, DNA fragmentation and histopathological data over a period of 90 days. Mol. Cell. Biochem..

[23] Allen T.M., Cullis P.R. (2013). Liposomal drug delivery systems: From concept to clinical applications. Adv. Drug Deliv. Rev..

[24] Bozzuto G., Molinari A. (2015). Liposomes as nanomedical devices. Int. J. Nanomed..

[25] Caddeo C., Manconi M., Sinico C., Valenti D., Celia C., Monduzzi M., Fadda A.M. (2015). Penetration Enhancer-Containing Vesicles: Does the Penetration Enhancer Structure Affect Topical Drug Delivery?. Curr. Drug Targets.

[26] Caddeo C., Pucci L., Gabriele M., Carbone C., Fernandez-Busquets X., Valenti D., Pons R., Vassallo A., Fadda A.M., Manconi M. (2018). Stability, biocompatibility and antioxidant activity of PEG-modified liposomes containing resveratrol. Int. J. Pharm..

[27] Sercombe L., Veerati T., Moheimani F., Wu S.Y., Sood A.K., Hua S. (2015). Advances and Challenges of Liposome Assisted Drug Delivery. Front. Pharmacol..

[28] Ezhilarasi P.N., Muthukumar S.P., Anandharamakrishnan C. (2016). Solid lipid nanoparticle enhances bioavailability of hydroxycitric acid compared to a microparticle delivery system. RSC Adv..

